# Effect of A Fluoride Toothpaste Containing Enzymes and Salivary Proteins on Periodontal Pathogens in Subjects with Black Stain: A Pilot Study

**DOI:** 10.1055/s-0043-1761193

**Published:** 2023-03-04

**Authors:** Maria Carelli, Iuliia Zatochna, Angela Sandri, Gloria Burlacchini, Angelica Rosa, Francesca Baccini, Caterina Signoretto

**Affiliations:** 1 Department of Diagnostics and Public Health, Microbiology Section, University of Verona, Verona, Italy; 2 Dental Hygiene Unit, Azienda Provinciale per i Servizi Sanitari of Trento, Rovereto, Italy

**Keywords:** black stain, enzyme-based toothpaste, oral hygiene, periodontal pathogens.

## Abstract

**Objective** Black stain (BS) is an extrinsic dental discoloration particularly difficult to treat. Although its etiology is not fully clear yet, chromogenic bacteria inside the oral cavity seem to be involved. In this pilot study, we evaluated whether a toothpaste containing enzymes and salivary proteins could improve oral health and reduce the presence of periodontal pathogens in subjects predisposed to BS discoloration.

**Materials and Methods** Twenty-six subjects were enrolled in the study: 10 subjects without BS; 16 subjects with BS, randomly assigned in two groups: test ( *n* = 8) and control ( *n* = 8). The test group used a toothpaste containing sodium fluoride, enzymes, and salivary proteins. The control group used a toothpaste with amine fluoride. At enrollment and after 14 weeks, participants were subjected to professional oral hygiene, evaluation of BS (through Shourie index) and oral health status, collection of saliva and dental plaque samples. The presence of periodontal pathogens in plaque and saliva of all subjects was investigated by molecular analysis (PCR).

**Statistical Analysis** The prevalence of investigated microbial species in patients with/without BS was performed by Chi-squared test. The variation in the prevalence of the investigated species after treatment in test and control group was analyzed by *t* -test.

**Results** Clinical evaluation showed that 86% of participants with BS had a reduction in the Shourie index, independently from the toothpaste used. In particular, a greater reduction in the Shourie index was observed in subjects using an electric toothbrush. We did not observe an effect of the fluoride toothpaste containing enzymes and salivary proteins on the composition of the oral microbiota of the test subjects in comparison with controls. When comparing all subjects with BS ( *n* = 16) and without BS ( *n* = 10), *P. gingivalis* detection was significantly higher in saliva samples collected from subjects with BS ( *p* = 0.0129).

**Conclusion** We verified that the use of an enzyme-containing toothpaste alone is not sufficient to prevent the formation of BS dental pigmentation in subjects predisposed to this discoloration. Mechanical cleaning, especially using electrical toothbrushes, seems to be useful to counteract BS formation. Moreover, our results suggest a possible association between BS and the presence of *P. gingivalis* at the salivary level.

## Introduction

Black stain (BS) is a specific type of extrinsic dental dyschromia. 1 It was described by Wilkins in 2005 as dark spots with a linear appearance or points of incomplete coalescence. They rarely extend beyond the cervical third of the crown but may also affect the base of the grooves and dental pits. 2 Koch et al defines BS as a continuous or dashed black line on the surface of the tooth that follows the gingival margin. 3 BS has a reported prevalence between 1% and 20% and can occur at any age although its prevalence appears to peak in childhood. 4 In young subjects, BS tends to regress with pubertal development and transition to adult life. 5 Studies have shown equal prevalence in both sexes 4 although there is a report of greater predisposition in women. 6

**Fig. 1:**
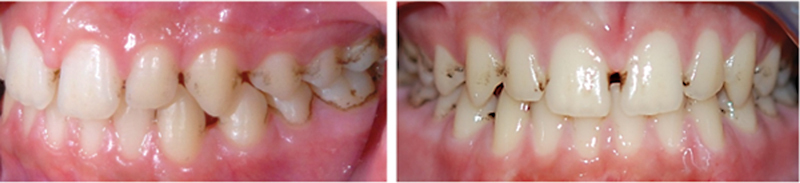
Photograph of teeth affected by BS (subject P05).

Although the causative factors of BS are not fully understood, the presence of chromogenic bacteria inside the oral cavity seems to be involved. 7 8 These bacteria can produce iron-binding substances such as hydrogen sulfide suggesting that sulfur and metal ions, normally free, inside the oral cavity may form an insoluble intensely-colored compound that precipitates on the teeth 9 ( Fig. 1 ). Putative bacteria involved in BS formation are *Porphyromonas gingivalis, Prevotella intermedia, Aggregatibacter actinomycetemcomitans, Tannerella forsythia, Treponema denticola, Actinomyces naeslundii, Actinomyces* spp., and *Veillonella* spp. Interestingly, some studies have shown that subjects suffering from BS are less prone to caries. 10 11 12 13

Few scientific data are available on BS and there are no defined protocols for the management of patients with this type of dyschromia, which cannot be removed with the normal procedures of home oral hygiene. Electric brushing can help improving the overall plaque score but no report on removal of BS is available. 14 Professional hygiene is a temporary solution but it does not address the real causes of the formation of BS pigments. Effective treatment of BS still represents a challenge for dental hygienists. 15 Therefore, alternative effective strategies for its management are needed. Adams et al demonstrated that brushing with a toothpaste containing enzymes and proteins promotes a shift in the ecology of the oral microbial community over time in healthy subjects, increasing natural salivary defenses. This general change in the bacterial community seems to be associated with oral health due to the consequential increase of bacteria favoring gum health and the concomitant decrease of bacteria causing periodontal diseases such as BS. 16 17 18 19

Following these results obtained in healthy subjects, we aimed at evaluating the effects of a fluoride toothpaste containing enzymes and salivary proteins on periodontal pathogens associated with BS. A secondary objective was to define the indications for the management of patient with this extrinsic pigmentation.

## Materials and Methods

### Pilot Study Design

Twenty-six subjects were enrolled in the study: 10 subjects without BS and 16 subjects with BS. Subject with BS were randomly assigned in two groups of equal number: test ( *n* = 8) and control ( *n* = 8). The test group used a toothpaste containing sodium fluoride, enzymes, and salivary proteins; the control group used a toothpaste with amine fluoride (see subsection “toothpastes used in the study”). Both test and control groups used the assigned toothpaste twice a day for 14 weeks. The pilot study was double-blinded; sequentially numbered, opaque, sealed envelopes containing the test or the control toothpaste were prepared.

At the beginning of the study (T0), all subjects completed a questionnaire including questions regarding socio-demographic variables such as age and sex. Subjects with BS (test and control groups) were also asked about oral hygiene habits such as type of dental brush (manual or electric) and dietary habits such as iron supplementation and type of drinking water consumed. Inclusion criteria: subjects m/f aged from 6 to 50 years with negative medical dental history. Exclusion criterion: increased consumption of iron, e.g., frequent consumption of iron supplements, tap water, cheese, and red meat (other causes of BS).

The test and control subjects received training for home dental hygiene at enrollment (T0) and were contacted by phone by a dental hygienist every 2 weeks to ensure adherence to the protocol. Fourteen weeks later (T1), participants of test and control groups provided their feedback on the protocol through a questionnaire.

All enrolled subjects were followed at the Dental Hygiene Unit, Azienda Provinciale per i Servizi Sanitari of Trento, Rovereto, Italy. The study was consistent with good clinical practices in accordance with the Ethical Principles of the 64th World Medical Association Declaration of Helsinki. All participants signed a written informed consent.

### Toothpastes Used in the Study

The test group used a toothpaste containing 1450 ppm sodium fluoride, enzymes, and salivary proteins (Zendium; ingredients: Aqua, Hydrated Silica, Sorbitol, Glycerin, Steareth-30, Xanthan Gum, Aroma, Carrageenan, Disodium Phosphate, Sodium Fluoride, Amyloglucosidase, Citric Acid, Zinc Gluconate, Sodium Benzoate, Glucose Oxidase, Sodium Saccharin, Potassium Thiocyanate, Colostrum, Lysozyme, Lactoferrin, Lactoperoxidase). The control group used a toothpaste with 1450 ppm amine fluoride (Elmex; Aqua, Hydrated Silica, Sorbitol, Hydroxyethylcellulose, Olaflur, Aroma, Saccharin, Limonene CI 77891).

### Clinical Examination and Samples Collection

All subjects with BS (both test and control group) underwent intraoral examination and professional oral hygiene at the enrollment (T0) and 14 weeks later (T1), performed by the same dental hygienist. Moreover, intraoral photographs were collected. Clinical evaluation of BS was assessed trough the Shourie index (numerical index of BS pigmentation used as criterion for differential diagnosis of BS). Each patient's BS score was assigned as follows: 1 = no black spots, 2 = incomplete lines of pigmented spots, 3 = pigmented solid lines. Oral health status of each participant was assessed by calculating Plaque Control Record index (O'Leary), Bleeding Ginvival Index (GBI, Ainamo & Bay), Mean number of Decayed, Missing, and Filled primary and permanent Teeth index (DMFT/dmft).

### Samples Collection

Subjects of test and control groups underwent collection of plaque and saliva samples at the enrollment (T0) and 14 weeks later (T1). Collection of plaque and saliva from negative control group was performed only at enrolment (T0). About 1 mg of the supragingival dental plaque was collected by scraping with a plastic scaler from tooth surfaces, transferred to sterile endodontic paper points and stored at −20°C in sterile DNA- and RNA-free Eppendorf tubes containing TE buffer (Tris-EDTA). In total, 4 mL of unstimulated whole saliva samples were collected by spitting in a sterile tube.

### Molecular Analysis

Bacterial DNA was extracted from plaque and saliva samples using GenElute Bacterial Genomic DNA Kits (Sigma-Aldrich). Two different multiplex PCR were performed to identify the presence of *P. gingivalis, P. intermedia* , and *A. actinomycetemcomitans* , 20 and *T. forsythia, T. denticola, A. naeslundii* . 21 Additionally, single PCR was performed to identify *Actinomyces* spp, 22 *Veillonella* spp. 23 and *S. mutans* . 24 Primers and PCR conditions are reported in Supplementary Table S1 , available online only. PCR reaction was performed using the 5Prime Hot Master Mix (Quantabio) according to manufacturer's instructions.

**Table 1:** Demographic characteristics and hygiene habits of participants

Group		Test	Control	Negative control
Age (mean ± SD)		22.5 ± 12.7	19.8 ± 15	12.9 ± 5.7
Gender	Male	2	3	4
	Female	6	5	6
BS	Yes	8	8	0
	No	0	0	10
Toothbrush	Electric	2	3	NA
	Manual	6	5	NA

### Statistical Analysis

Statistical analysis of the prevalence of the investigated microbial species in patients with and without BS was performed by Chi-squared test. The variation in the prevalence of the investigated species after treatment in test and control groups was analyzed by *t* -test. Significance level was set at 0.05. All analyses were performed using GraphPad Prism 7.0.

## Results

A total of 16 volunteers with BS and 10 subjects without BS were recruited in the study. Subjects with BS were divided into two groups, test ( *n* = 8) and control ( *n* = 8), depending on the toothpaste that they were assigned to use during the study. The mean age was 22.5 ± 12.7 years for the test group, 19.8 ± 15 years for the control group, and 19.9 ± 5.7 years for volunteers without BS ( Table 1 ). Age showed no association with the presence of BS. Although female participants with BS were 11 versus 5 males, sex cannot be considered a risk factor for BS due to the small sample size. Participants were asked about their oral hygiene: we found no difference in BS appearance related to the type of dental brush daily employed. As concerns dietary habits, none of the subjects enrolled in the study reported to consume daily iron supplements, tap water, cheese, or red meat (exclusion criteria). Two subjects in the test group dropped out of the pilot study due to poor compliance.

### Evaluation of Oral Health in Subjects with BS

Oral health status of the BS subjects was assessed at T0 using the DMFT/dmft index, GBI and Plaque Control Record ( Table 2 ). Interestingly, a high number of subjects with BS (56%) also presented decayed, missing, and filled primary and permanent teeth (DMFT/dmft > 1, median = 0.5).

**Table 2:** Assessment of risk factors related to the oral health status of the participants, including DMFT/dmft index, Plaque Control Record index (%) and gingival bleeding (GBI, %)

Group	Patient	DMFT/dmft index	Plaque control record (%)	GBI (%)
Test	P01	0	23	3
	P05	0	18	0
	P06	0	16	6
	P07	3	25	17
	P09	5	75	35
	P10	2	14	10
	P14	1	16	0
	P16	3	7	0
Control	P02	0	25	18
	P03	1	26	0
	P04	0	60	9
	P08	0	23	0
	P11	0	12	7
	P12	0	18	16
	P13	2	37	25
	P15	6	40	9

### Prevalence of Periodontal Pathogens

**Fig. 2:**
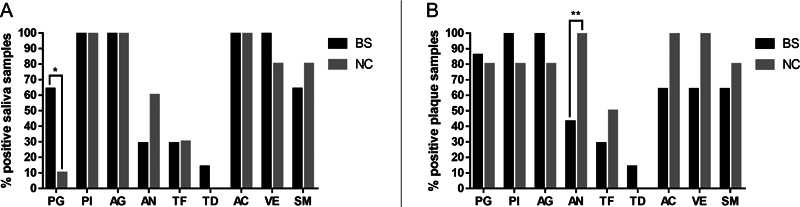
Prevalence (%) of the investigated bacteria present in saliva ( **A** ) and dental plaque ( **B** ) of patients with black stain (BS) and negative controls (CN) at T0. Statistical analysis was performed by Chi Squared test, * *p* < 0.05 and ** *p* < 0.01. PG = *P. gingivalis* ; PI = *P. intermedia* ; AG = *A. actinomycetemcomitans* ; AN = *A. naeslundii* ; TF = *T. forsythia* ; TD = *T. denticola* ; AC = *Actinomyces* spp.; VE = *Veillonella* spp.; SM = *S. mutans* .

A total of 26 saliva and dental plaque samples were collected at T0 from all enrolled participants (16 subjects with BS and 10 subjects without BS) and analyzed by PCR assay. We identified the presence of *P. intermedia, A. actinomycetemcomitans, Veillonella* spp. and *Actinomyces* spp. in all saliva samples from subjects with BS. Other bacterial species identified in saliva of subjects with BS were *P. gingivalis* (64%), *A. naeslundii* (29%), *T. forsythia* (29%), and *T. denticola* (10%). Equal or comparable prevalence was observed in their dental plaque ( Fig. 2 ). Interestingly, when comparing subjects with and without BS, *P. gingivalis* detection was significantly higher in saliva samples collected from subjects with BS ( *p* = 0.0129), while detection of *A. naeslundii* in their dental plaque was significantly lower ( *p* = 0.0064).

### Effects of Enzymes and Salivary Proteins on Periodontal Pathogens

**Fig. 3:**
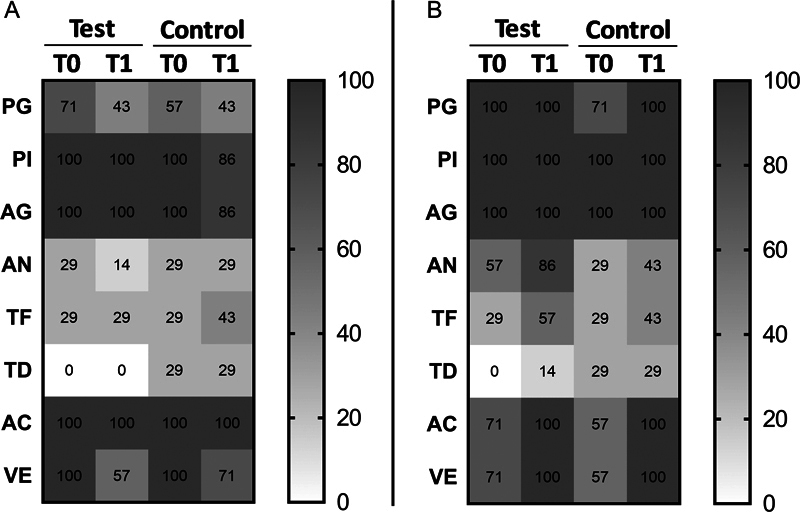
Prevalence (%) of the investigated bacteria present in saliva ( **A** ) and dental plaque ( **B** ) of test and control patients at T0 and T1. PG = *P. gingivalis* ; PI = *P. intermedia* ; AG = *A. actinomycetemcomitans* ; AN = *A. naeslundii* ; TF = *T. forsythia* ; TD = *T. denticola* ; AC = *Actinomyces* spp.; VE = *Veillonella* spp.

Within subjects with BS, the test group ( *n* = 8) used twice a day for 14 weeks a toothpaste containing 1450 ppm sodium fluoride, enzymes and salivary proteins, while the control group ( *n* = 8) used a toothpaste with 1450 ppm amine fluoride. At T1, 14 saliva and dental plaque samples were collected from test and control participants (two subjects were excluded due to poor compliance) and analyzed by PCR assay. Although no statistically significant difference regarding the investigated bacteria was found between T0 and T1 in the two groups, the test subjects showed a higher reduction of *P. gingivalis, A. naeslundii* and *Veillonella* spp. in their saliva with respect to the control subjects ( Fig. 3 ).

### Effects of Enzymes and Salivary Proteins on Oral Health

**Fig. 4:**
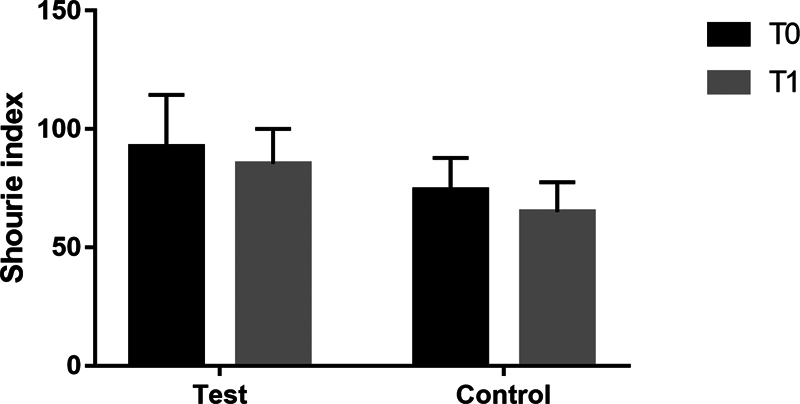
Shourie Index of the test group and the control group at T0 and at T1. Each bar shows the mean ± SD of each group of patients.

At T1, Shourie index showed a slight reduction in both test and control group (8% and 12% average reduction, respectively) although the difference was not statistically significant ( Fig. 4 ). In total, 86% of BS patients presented a reduction in their Shourie index ( Table 3 ). In particular, the reduction was slightly higher in patients using an electrical toothbrush than in those using a manual one.

**Table 3:** Shourie index of the participants with BS at T0 and T1

Group	Patient	Shourie index		Shourie index variation (%)
		T0	T1	
Test	P01	90	89	–1.1%
	P05	126	108	–14.3%
	P06 ^a,b^	NA	NA	NA
	P07 ^a^	100	78	–22.0%
	P09	58	63	+8.6%
	P10	94	88	–6.4%
	P14	87	86	–1.1%
	P16 ^b^	NA	NA	NA
Control	P02	71	67	–5.6%
	P03	54	54	0%
	P04 ^a^	69	50	–27.5%
	P08 ^a^	61	50	–18.0%
	P11	71	70	–1.4%
	P12 ^a^	92	74	–19.6%
	P13	87	68	–21.8%
	P15	88	86	–2.3%

Notes: ^a^ Indicates patients using an electric toothbrush; ^b^ indicates dropouts (due to poor compliance).

PCR; TD, *T. denticola* ; TF, *T. forsythia* ; VE, *Veillonella* spp.

## Discussion

Recent studies have highlighted that toothpastes containing enzymes and salivary proteins may influence oral microbiome, viability of oral bacteria, and gingival health, inducing the increase of bacteria favoring oral health while reducing others causing oral problems. 16 17 18 19 We tested this hypothesis in a pilot study with 16 subjects affected by BS. Similar to previous studies, 16 18 we compared subjects using a fluoride toothpaste containing enzymes and salivary proteins with subjects using a standard fluoride toothpaste (without enzymes and proteins) for 14 weeks. Noteworthily, in addition to plaque microbiota, we also evaluated saliva microbiota to obtain a complete assessment of the oral microbiota. The microbiological composition of BS is thought to be dominated by *Actinomycetes* including *A. naeslundii* and other *Actynomices* species, as well as gram-negative anaerobic bacteria such as *T. forsithya* , *T. denticola* , *P. gingivalis, A. actinomycetemcomitans, P. intermedia,* and *Veillonella* spp. Therefore, alterations in the presence of these periodontal pathogens, both in plaque and/or in saliva, could be associated with BS formation.

From our results, the use of a toothpaste containing enzymes and salivary proteins for 14 weeks did not produce significant effects on the composition of the oral microbiota nor on oral health of the test subjects in comparison with controls. The reduction in some pathogenic bacteria associated with BS was observed in saliva of test subjects, but not in their dental plaque. This may lead to hypothesize that the enzymes and proteins contained in the toothpaste could have a partial efficacy against salivary planktonic bacteria, but little or no activity on the bacteria that form the plaque biofilm. This can be explained by the fact that salivary bacteria are much more susceptible to enzymatic action than those in the dental plaque, where the outer layer of exopolysaccharide prevents the entry and diffusion of various substances within the biofilm. 25 As our study is limited to a small sample of 16 patients with BS followed for a short time of 14 weeks, further studies on a larger sample and monitored for a longer period of time would be needed to evaluate possible changes in the microbial composition of the dental plaque.

When comparing oral bacteria between subjects with and without BS, major differences were observed only for *A. naeslundii* and *P. gingivalis* . The latter showed a significantly higher prevalence in saliva samples collected from subjects with BS, while *A. naeslundii* resulted more prevalent in the plaque of subjects without the discoloration. This last observation is in contrast with previous studies reporting higher abundance of *A. naeslundii* in the dental plaque of subjects with BS compared with BS-free subjects. 12 26 However, another study reported a similar prevalence of *Actinomyces* spp. in patients with BS and controls. 27 As for *P. gingivalis* , a previous study reported its absence in BS deposits, but no data were available regarding saliva of subjects with BS. 15 As saliva is the medium for planktonic suspension in the oral environment, it is in strict relation with and likely contributes to formation of the plaque biofilm. Many studies also highlighted the association of saliva microbiota and oral health-related conditions. 28 29 As such, studies evaluating the involvement of both saliva and plaque microbiota should be performed to identify potential etiological agent associated with BS.

Most studies also report a correlation between the presence of BS and lower caries experience. Conversely, in our study a high number of subjects with BS (56%) also presented decayed, filled or extracted for caries teeth. Moreover, we observed no significant difference in the prevalence of *S. mutans* between subjects with and without BS, similarly to what previously reported by Costa et al. 27 Although this is apparently in contrast with most literature, other factors such as dietary habits, socioeconomic status, and iron supplementation may contribute to the formation of BS in addition to alterations of the oral microflora.

At a clinical level, 14 weeks after enrollment 86% of participants with BS showed a reduction in the Shourie index-numerical index of black stain pigmentations, as well as a criterion in the differential diagnosis of black stains–independently from the toothpaste used. This result shows that the mechanical removal through meticulous home oral hygiene procedures is of primary importance in the removal of BS. Moreover, subjects using an electric toothbrush showed a greater reduction in the Shourie index when compared with the average reduction achieved by patients who used the manual toothbrush. Although this observation has no statistical significance due to imbalanced number of subjects using electric and manual toothbrush, it may support previous scientific evidence reporting higher efficacy of the electric toothbrush in removing plaque, food residue, and extrinsic stains probably due to the much faster rocking motions achieved in comparison to manual brushing. 14

## Conclusions

We verified that the use of a toothpaste containing enzymes and salivary proteins alone is not sufficient to prevent the formation of BS in subjects predisposed to this discoloration. Thus, mechanical cleaning, especially using electrical toothbrushes, remains the primary care to counteract BS formation. Additionally, it was possible to highlight the presence of *P. gingivalis* at the salivary level as a potential etiological agent associated with BS, further underlining the complexity of the microbiological community that could be involved in BS formation and persistence. However, the size of our pilot study was limited to 16 participants with large age span; to have a clearer and more complete vision, the cohort of patients to be enrolled should be expanded to reach a more consistent size. Nonetheless, these results could be of interest for dental health professionals dealing with BS in their clinical routine and searching for new solutions to support their patients' dental health.
