# Supplementary material for: Effect of A Fluoride Toothpaste Containing Enzymes and Salivary Proteins on Periodontal Pathogens in Subjects with Black Stain: A Pilot Study
Source: Eur J Dent. 2023 Mar 4;18(1):109–16. doi: 10.1055/s-0043-1761193 (PMC10959611; doi:10.1055/s-0043-1761193)
Supplement: Supplementary file 1 — Supplementary Material [file 10-1055-s-0043-1761193-s22102427.pdf]

Supplementary Table S1 Primers and PCR conditions used in the pilot study

Type	Target	Sequence	Reference	PCR type	Annealing
F	16s rRNA	CGT GCC AGC AGC CGC GGT AAT ACG	Garcia et al., 1998 (9)	mPCR	70°C
R	PI	TCC GCA TAC GTT GCG TGC ACT CAA G			
R	PG	TAC ATA GAA GCC CCG AAG GAA GAC G			
R	AG	CTT TGC ACA TCA GCG TCA GTA CAT CCC CAA GG			
F	TD	GCA AGA CTT GTA GCG GTA GT	Pardo et al., 2021 (21)	mPCR	60°C
R	TD	GAT GCC TAT TTG CGG GCT TG			
F	TF	CGG TGG TCT CCA ATC TCA CC			
R	TF	GCC CTC AAC ACA CGA CAC TT			
F	AN	GGA ATG ATG GCG TGA ATG GC			
R	AN	CCG ATC CCG TGA GTA CAT GG			
F	AC	GGC KTG CGG TGG GTA CGGG C	Xia et al., 2003 (22)	sPCR	60°C
R	AC	GGC TTT AAG GGA TTC GCT CCR CCT CAC			
F	VE	GTA ACA AAG GTG TCG TTT CTC G	Mashima et al., 2016 (23)	sPCR	60°C
R	VE	GCA CCR TCA AAT ACA GGT GTA GC			
F	SM	GGCACCACAACATTGGGAAGCTCAGTT	Nakano et al., 2006 (24)	sPCR	70°C
R	SM	GGAATGGCCGCTAAGTCAACAGGAT			

Abbreviations: AC, *Actinomyces* spp.; AG, *A. actinomycetemcomitans*; AN, *A. naeslundii*; F, forward primer; mPCR, multiple PCR; PG, *P. gingivalis*; PI, *P. intermedia*; R, reverse primer; SM = *S. mutans*.sPCR, single

 © 2023. The Author(s).
